# Advantage of laparoscopic peritoneal toileting in acute peritonitis with unclear etiology: A case report with inspiring outcome (with video)

**DOI:** 10.1016/j.ijscr.2020.05.089

**Published:** 2020-06-12

**Authors:** Md. Sumon Rahman, Hasan Ul Banna, Md. Nazmul Hasan, Mohammad Jumman

**Affiliations:** a Department of Surgery, Dhaka Community Medical College Hospital, Dhaka, Bangladesh; b Department of Surgery, Ibn Sina Medical College Hospital, Dhaka, Bangladesh; c Sylhet MAG Osmani Medical College, Sylhet, Bangladesh; d Department of Surgery, Jahurul Islam Medical College Hospital, Bajitpur, Bangladesh

**Keywords:** Peritonitis, Laparotomy, Laparoscopy, Case report

## Abstract

**Introduction:**

Acute peritonitis is a surgical emergency and is conventionally managed with exploratory laparotomy in many centers, but laparoscopy is not contraindicated in such conditions. Operative management remains a cornerstone treatment in peritonitis irrespective of the primary pathology.

**Presentation of case:**

A 32-years-old man was admitted with acute diffuse peritonitis. As he was clinically stable, we proceeded with conservative treatment and diagnostic workup. He did not respond by 24 h and CT imaging showed ascites, but no definite diagnosis could be made. Diagnostic laparoscopy was performed, thorough peritoneal toileting was done but surprisingly the etiology was unclear. Following laparoscopy, he responded well with an uneventful recovery.

**Discussion:**

Timely proper evaluation and correct intervention are a paramount concern for the positive outcome. Regarding the most effective operative approach (laparotomy or laparoscopy) in acute peritonitis remains controversial in the literature and laparoscopy has been considered as a relative contraindication. However, the laparoscopy is gaining wider acceptance as the limitations of laparoscopy are declining rapidly. We experienced a case of extensive peritonitis with unclear etiology which we managed successfully by laparoscopy.

**Conclusion:**

Laparoscopic approach in peritonitis is effective and helpful for accurate diagnosis and to minimize the hazards of an unnecessary laparotomy. This case report might encourage many surgeons to consider laparoscopic approach in peritonitis with confidence and to perform further studies.

## Highlights

• Clinical practice guideline by EAES suggests, laparoscopy is not contraindicated in peritonitis.
• Etiology of acute peritonitis is unclear in some cases.
• Laparoscopy under local anaesthesia can be done. The cause of peritonitis might be missed in conventional imaging.
• Peritoneal toileting is more efficiently done by laparoscopy.
• Diagnostic laparoscopy may avoid the hazards of unnecessary laparotomy.

## 1 Introduction

Peritonitis refers to the peritoneal inflammation and a common cause of surgical emergency which was traditionally managed by exploratory laparotomy. Peritonitis may be classified as primary spontaneous peritonitis and secondary peritonitis. Primary spontaneous peritonitis includes the risk factors like cirrhotic liver disease with ascites and renal failure needs dialysis. In primary peritonitis, previous medical history and medications are quite suggestive. Secondary peritonitis may follow any abdominal infection such as appendicitis, perforation of hollow viscus, typhoid or tubercular ulcer of gastrointestinal tract, pancreatitis, inflammatory bowel disease, pelvic inflammatory disease, urinary tract infection or abdominal trauma etc. The paramount concern in management is to relieve the patient from the toxins and bacterial loads from the abdominal cavity and control of the source as early as possible. Any improper evaluation and interventional delay might have a fatal outcome. The patient should be hospitalized immediately and treated with broad spectrum intravenous antibiotics, parenteral fluid, and nutritional support. Commonly encountered surgical indications of peritonitis include ruptured appendix, perforated duodenal ulcer, colonic diverticulitis, typhoid ulcer perforation, ischemic colitis, pelvic inflammatory disease and so on [[1], [2], [3]].

Emergency exploration is indicated to control the source of sepsis even in diagnostic uncertainty. In this regard, the value of laparoscopy has been recognized since 1950. Laparoscopy under local anaesthesia is also useful in acute peritonitis to visualize the whole abdomen and pelvis providing the diagnosis which might be missed in conventional imaging [4,5]. Even after exploratory laparotomy the etiology may remain unclear and could results in additional morbidity and mortality. The diagnostic accuracy of laparoscopy has been reported to be almost 90 percent [6,7], but Kirshtein reported it as higher as 98 percent [8].

This is a report on a single patient with etiological uncertainty and its outcome of interest with laparoscopic surgery. We managed our patient in Jahurul Islam Medical College Hospital, a tertiary care center at a remote area in February 2019. This work was reported in line with the SCARE criteria [9].

## 2 Case presentation

A 32-years-old man was admitted with the complaints of severe and diffuse abdominal pain associated with mild fever for last 8 h. He had no bowel movement within last 24 h and respiratory discomfort for one and half an hour. He was hemodynamically stable with optimum oxygen saturation and temperature was recorded 99 °F with some signs of dehydration. His abdomen was not distended but it was tender all over and rigid and digital rectal examination revealed normal. Treatment started with intravenous crystalloid infusion with antibiotics (cefoperazone + metronidazole) and analgesics.

**Fig. 1:**
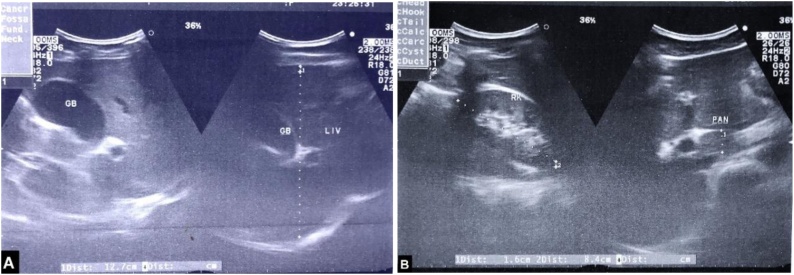
Image-A shows sonographic appearance of liver and gallbladder, image-B shows right kidney and pancreatic regions.

**Fig. 2:**
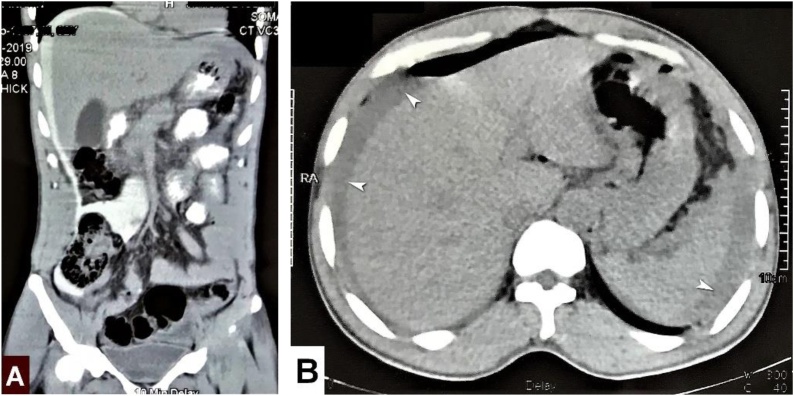
A & B shows huge peritoneal collection, white arrows in image-B.

Plain chest and abdominal radiograph with abdominal ultrasound (Fig. 1) appeared normal. Hematological and biochemical parameters revealed neutrophilia, mildly raised serum lipase, normal liver and renal function, and no electrolyte imbalance (Table 1). Urine analysis showed insignificant pyuria. On the 2nd day he had no significant improvement. So, abdominal CT was done that showed a significant amount of collection in the right sub-phrenic, sub diaphragmatic, and pelvic area (Fig. 2).

**Table 1:** Hematological and biochemical studies.

Hemoglobin	13 gm/dl
HCT	39.8%
TC of WBC	10,500/cu mm
Neutrophil	86.2%
Lymphocyte	11%
Platelet	230 K/cu mm
ESR	14 mm in 1^st^ hr
Creatinine	0.7 mg/dl
Lipase	62.6 U/L
Na^+^	142 mmol/L
K^+^	3.99 mmol/L
Cl^−^	105.4 mmol/L
HCO_3_	21.9 mmol/L
pH	7.33

**Fig. 3:**
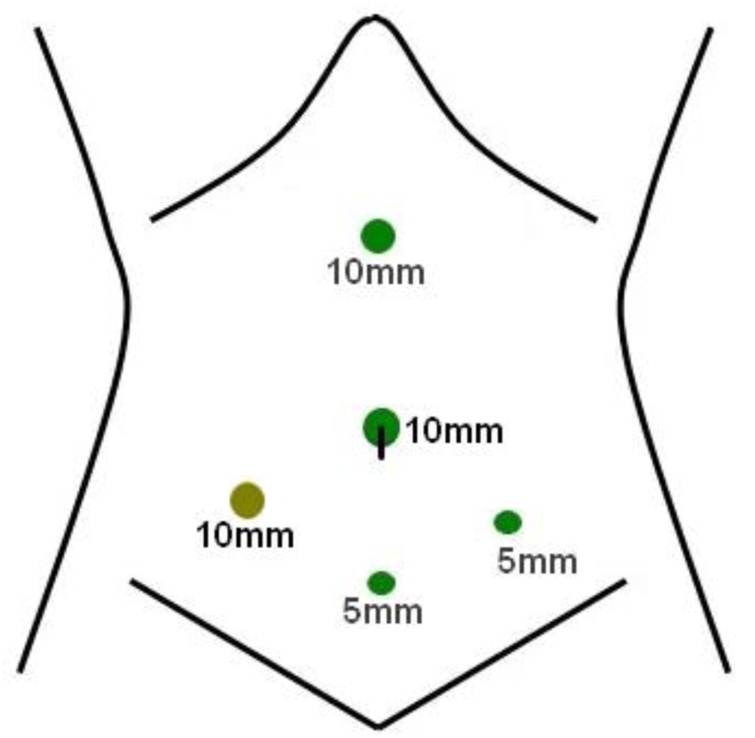
Laparoscopic ports placement.

**Fig. 4:**
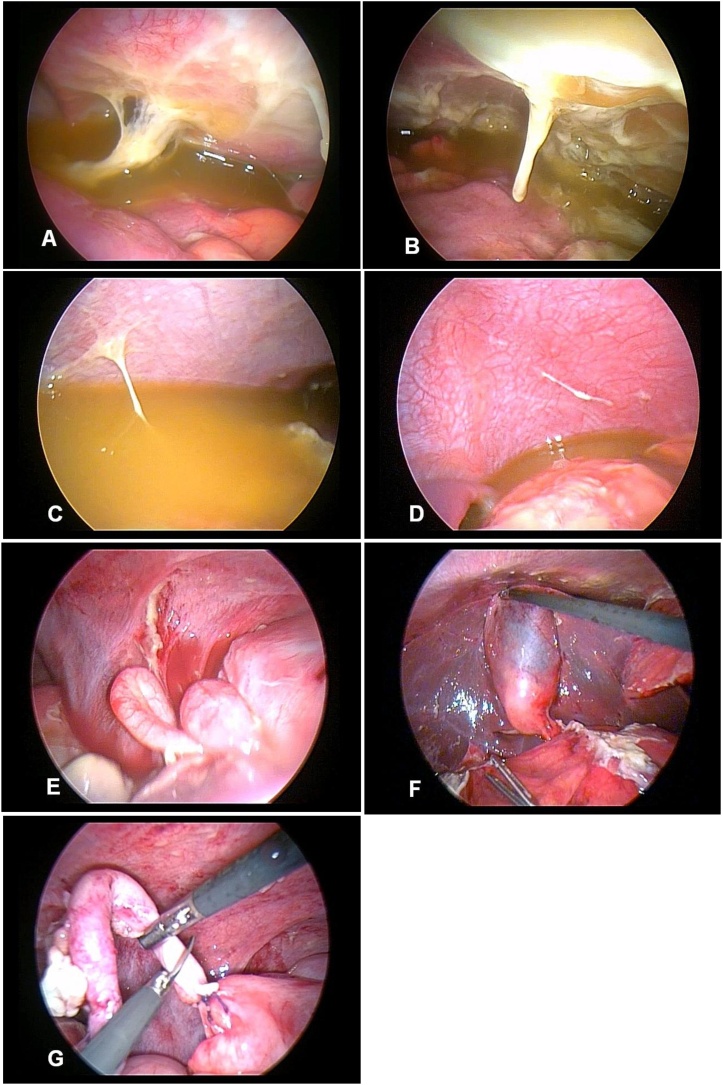
A: Right iliac fossa, B: Pelvis, C: Right upper quadrant, D: Left upper quadrant, E: Appendix, F: Gall bladder, G: Appendicectomy.

**Fig. 5:**
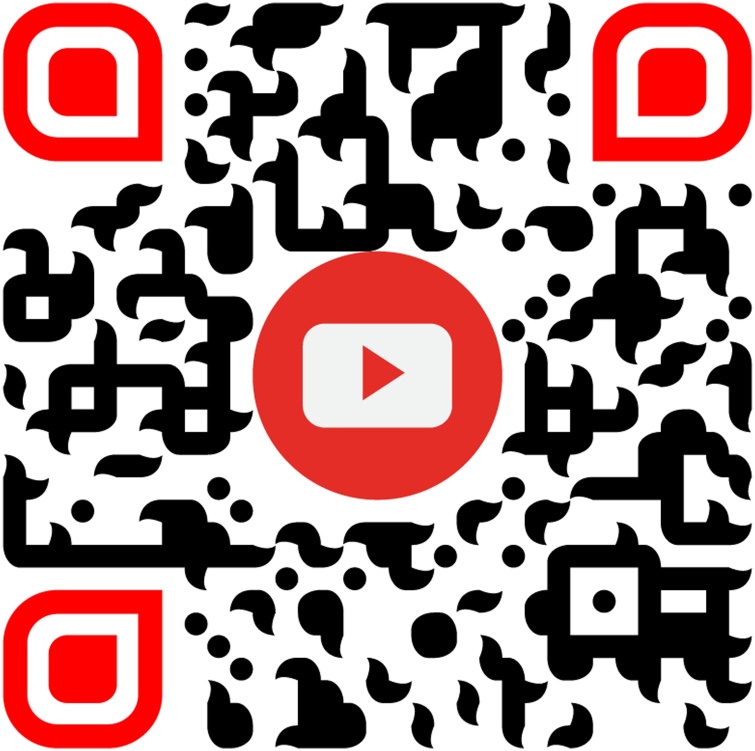
QR for video link.

Diagnostic laparoscopy proceeded with modified direct initial trocar access through umbilicus as our routine practice and other ports were placed as needed (Fig. 3). Huge amount of purulent and turbid collection was noted in all potential spaces (Fig. 4; A–D) with extensive fibrinous exudates over the liver, loops of intestine, and in the pelvic cavity. There was no organized lump in the abdomen and small bowel loops were mildly distended. The appendix was found swollen but no perforation (Fig. 4; E). Peritoneal fluids were sucked out and fibrinous exudates were swept out with gauze piece as much as possible. Peritoneal fluid was sent for culture and sensitivity test. Gastro-duodenal area, gallbladder (Fig. 4; F), and ileum were carefully inspected. The gastro-duodenal area was covered with dens exudates, but no bile leak was noted in closed magnified view. Appendicectomy was performed (Fig. 4; E, G) followed by thorough peritoneal irrigation with 2 L of warm normal saline and finally, a drain tube (DT) was placed in pelvis. Operative time was 90 min (Fig. 5; QR for video link to the https://youtu.be/DvZvXl20-ns). Anesthetic recovery was uneventful. The patient had symptomatic improvement on first post-operative day (POD) and DT collection was noted 150 mL, which gradually declined. Bowel sound appeared on 3rd POD and oral feeding resumed. Peritoneal fluid culture revealed no growth. Histopathology of the appendix revealed acute appendicitis. He was discharged with DT on 4th POD and did not develop any further complications like recurrent abdominal pain, intestinal obstruction, or any abdominal sepsis within 1 year of post-operative follow-up.

## 3 Discussion

Exploratory Laparotomy has been the mainstay of surgical management in acute peritonitis. And laparoscopy had been considered relatively contraindicated because of poor vision, inadequate peritoneal toileting, increased sepsis, time-consuming, post-operative adhesions, and late complications. But the recent trend is inclining more towards the laparoscopic management in acute peritonitis. We shall discuss the diagnostic dilemma and laparoscopic management outcome with our case report.

The medical history of our patient was not suggestive of primary peritonitis. The accurate diagnosis was crucial because many abdominal pathologies may mimic a similar presentation. Lactate, C-reactive protein are the well-known biomarkers of inflammation and raised in acute abdomen. But these are nonspecific and may mislead the diagnosis and require other unnecessary invasive interventions [10]. The conventional imaging may miss the diagnosis in acute peritonitis [4,5]. The diagnostic accuracy of abdominal ultrasound has been reported 60–89% and the CT abdomen is 84–98% [11,12]. In our case, conventional imaging revealed no diagnosis and CT scan detected only intraperitoneal collection. International literatures recommend the diagnostic accuracy of laparoscopy which would provide an adequate survey inside the abdomen for additional pathology [12].

A few inconsistent data mentioned increased abdominal pressure (IAP) during pneumoperitoneum seems to promote bacteremia [13]. But different experimental studies reported a higher risk of bacteremia, toxemia, and systemic inflammatory rate following laparotomy than laparoscopy in patients with acute peritonitis [[13], [14], [15]]. The clinical practice guideline by EAES, published in 2002 reported: *“…. there are no contraindications to create pneumoperitoneum when laparoscopic surgery is applicable in cases of peritonitis (grade B).”* The results of animal studies regarding the influence of pneumoperitoneum on bacteremia and endotoxemia are controversial [16]. Timely intervention is an important issue that greatly influences the treatment outcome. The severity of inflammation increases with time. *Argesta F. et al* documented 95% success rate with laparoscopic intervention underwent within 48 h of admission over 935 patients with acute peritonitis [17]. In our case, we performed laparoscopy within 48 h of admission following resuscitation and diagnostic workup.

The practical difficulties we used to encounter with laparoscopy in generalized peritonitis are difficult torcher access due to distended bowel loops, previous surgical scars, and peritoneal adhesions, and the possibility of iatrogenic injury, etc. Early conversion to open is essential in difficult cases. Laparoscopic management in patients with acute peritonitis recorded up to 23.3% conversion rate in a research article and was mainly due to intra-abdominal adhesions, obscured anatomy, and iatrogenic lesions [17].

Acute appendicitis is a disease mostly diagnosed clinically, yet investigations are advised accordingly to exclude other differential diagnoses. And any specimen retrieved from the abdomen with generalized peritonitis is expected to be infiltrated with acute inflammatory cells. Histologic appearance of acute appendicitis is obvious in this case but not seemed to be the etiology unless the appendix was gangrenous or perforated (Fig.4; E, G). So, the etiology remained unclear to us and very unlikely to produce such condition. However, we could manage this patient by laparoscopy and avoided an unnecessary laparotomy and its complications.

## 4 Conclusion

Clinical suspicion, the actual pathology, and the ultimate intervention are always subject to vary in acute abdominal conditions. In a suspected case of secondary peritonitis, laparoscopy could be attempted with the preparation of conversion to have the most accurate diagnosis and the best therapeutic intervention. And simultaneously the hazards of an unnecessary laparotomy could be avoided.

## Conflicts of interest

No conflict of interest.

## Funding

Solely funded by the authors.

## Ethical approval

This case reports the outcome of a routine management. Ethical Committee approval was not taken.

## Consent

Informed written consent was taken for scientific publication and presentation.

## Registration of research studies

1. Name of the registry: n/a

2. Unique identifying number or registration ID: n/a

3. Hyperlink to your specific registration (must be publicly accessible and will be checked): n/a

## Guarantor

Dr. Md. Sumon Rahman.

## Provenance and peer review

Not commissioned, externally peer-reviewed.

## CRediT authorship contribution statement

**Md. Sumon Rahman:** Conceptualization, Methodology, Data curation, Writing - original draft, Funding acquisition, Supervision, Validation. **Hasan Ul Banna:** Formal analysis, Writing - review & editing. **Md. Nazmul Hasan:** Data curation, Software. **Mohammad Jumman:** Data curation.

## References

[1] Sartelli M., Catena F., Di Saverio S., Ansaloni L., Malangoni M., Moore E.E. (2014). Current concept of abdominal sepsis: WSES position paper. World J. Emerg. Surg..

[2] Sartelli M., Catena F., Abu-Zidan F.M. (2017). Management of intra-abdominal infections: recommendations by the WSES 2016 consensus conference. World J. Emerg. Surg..

[3] Rhodes A., Evans L.E., Alhazzani W. (2017). Surviving Sepsis Campaign: International Guidelines for Management of Sepsis and Septic Shock: 2016. Intensive Care Med..

[4] Llanio R., Soto A., Ferret O., Gimenez G., Nordase O., Budapest O. (1976). Diagnostic De L’Abdomen Aigu Par Laparoscopie. Experience Portant 6400 Cas. 3 European Congress of Gastrointestinal Endoscopy. Abstract. June–July 1976.

[5] Llanio R., Sarle H. (1956). Interet de la peritoneoscope chez politraumatises. Marseille Chirurg..

[6] Nagy A.G., James D. (1989). Diagnostic laparoscopy. Am. J. Surg..

[7] Navez B., d’Udekem Y., Cambier E., Richir C., de Pierpont B., Guiot P. (1995). Laparoscopy for management of nontraumatic acute abdomen. World J. Surg..

[8] Kirshtein B., Roy-Shapira A., Lantsberg L., Mandel S., Avinoach E., Mizrahi S. (2003). The use of laparoscopy in abdominal emergencies. Surg. Endosc..

[9] Agha R.A., Borrelli M.R., Farwana R., Koshy K., Fowler A., Orgill D.P., For the SCARE group (2018). The SCARE 2018 statement: updating consensus surgical CAse REport (SCARE) guidelines. Int. J. Surg..

[10] Meyer Z.C., Schreinemakers J.M., van der Laan L. (2012). The value of C-reactive protein and lactate in the acute abdomen in the emergency department. World J. Emerg. Surg..

[11] Salem T.A., Molloy R.G., O’Dwyer P.J. (2005). Prospective study on the role of the CT scan in patients with an acute abdomen. Colorectal Dis..

[12] Sauerlenad S., Agresta F., Bergamaschi R., Borzellino G., Budzynsky A., Champault G., Fingerhut A., Isla A., Johansson M., Lundorff P., Navez B., Saad S., Neugebauer E.A. (2006). Laparoscopic for abdominal emergencies: evidence-based guidelines of the European Association for Endoscopic Surgery. Surg. Endosc..

[13] Fabian T.C., Croce M.A., Stewart R.M., Prichard F.E., Minard G., Kudsk K.A. (1993). A postoperative analysis of diagnostic laparoscopy in trauma. Ann. Surg..

[14] Blochle C., Rmmermann A., Strate T., Scheurlen U.J., Schineider C., Achilles E., Wolf M., Mack D., Zoring C., Broelsch C.E. (1998). Laparoscopic vs open repair of gastric perforation and abdominal lavage of associated peritonitis in pigs. Surg. Endosc..

[15] Narchi P., Benhamou D., Fernandez H. (1991). Intraperitoneal local anesthetic for shoulder pain after day case laparoscopy. Lancet.

[16] Neudecker J., Sauerland S., Neugebauer E. (2002). The European Association for Endoscopic Surgery clinical practice guideline on the pneumoperitoneum for laparoscopic surgery. Surg. Endosc..

[17] Agresta F., Ciardo L.F., Mazzarolo G. (2006). Peritonitis: laparoscopic approach. World J. Emerg. Surg..

